# Targeted Integration of siRNA against Porcine Cytomegalovirus (PCMV) Enhances the Resistance of Porcine Cells to PCMV

**DOI:** 10.3390/microorganisms12040837

**Published:** 2024-04-22

**Authors:** Hongzhen Mao, Jinyang Li, Mengyu Gao, Xinmei Liu, Haohan Zhang, Yijia Zhuang, Tianyi He, Wei Zuo, Lang Bai, Ji Bao

**Affiliations:** 1 Department of Pathology, Institute of Clinical Pathology, Key Laboratory of Transplant Engineering and Immunology, National Health Commission of China, West China Hospital, Sichuan University, Chengdu 610041, China; 2 Department of Pathology, Regeneration Medicine Research Center, West China Hospital, Sichuan University, Chengdu 610041, China; 3 Key Laboratory of Transplant Engineering and Immunology, National Health Commission of China, West China Hospital, Sichuan University, Chengdu 610041, China; 4 Department of Organ Regeneration, Shanghai East Hospital, Tongji University School of Medicine, Shanghai 200120, China; 5 Center of Infectious Diseases, West China Hospital, Sichuan University, Chengdu 610041, China

**Keywords:** porcine cytomegalovirus, CRISPR/Cas9, RNAi, knock-in, site-specific integration

## Abstract

In the world’s first pig-to-human cardiac cytomegalovirus (PCMV), xenotransplant and elevated levels of porcine key factors contributing to patient mortality were considered. This has renewed attention on PCMV, a virus widely prevalent in pigs. Currently, there are no effective drugs or vaccines targeting PCMV, and its high detection difficulty poses challenges for prevention and control research. In this study, antiviral small hairpin RNA (shRNA) was selected and inserted into the Rosa26 and miR-17-92 loci of pigs via a CRISPR/Cas9-mediated knock-in strategy. Further in vitro viral challenge experiments demonstrated that these genetically edited pig cells could effectively limit PCMV replication. Through this process, we constructed a PCMV-infected cell model, validated partial viral interference sites, enhanced gene knock-in efficiency, performed gene editing at two different gene loci, and ultimately demonstrated that RNA interference (RNAi) technology combined with CRISPR/Cas9 has the potential to generate pig cells with enhanced antiviral infection capabilities. This opens up possibilities for the future production of pig populations with antiviral functionalities.

## 1. Introduction

In January 2022, an innovative pig-to-human cardiac xenotransplantation in Maryland improved patient survival for two months post transplantation. However, elevated infection with porcine cytomegalovirus (PCMV) was detected and was considered one of the main factors responsible for the patient’s death [1]. PCMV belongs to the subfamily *Betaherpesvirinae* of the family *Herpesviridae*. It is an enveloped virus with a double-stranded linear DNA genome of approximately 230 kb containing more than 200 open reading frames encoding various proteins [2]. PCMV is widely distributed among pig populations [3]. Currently, there are no commercial vaccines targeting PCMV. The latent nature of PCMV, along with the difficulty in its detection, present challenges for prevention and control research [4]. However, the development of gene editing technologies offers a new approach for enhancing the biosafety of pigs [5]. By knocking in antiviral small interfering RNA (siRNA) sequences into the pig genome, PCMV replication can be continuously suppressed, thereby reducing the risk of viral infections. In recent years, studies have reported the possibility of reducing economic losses caused by Classical Swine Fever Virus (CSFV) by using CRISPR-Cas9 to insert anti-CSFV shRNA at specific sites [6]. This study employed CRISPR-Cas9 technology for gene editing, which has made significant advancements in gene knockout and knock-in [7]. The CRISPR-Cas9 system consists of the Cas9 nuclease and single-guide RNA (sgRNA), with the sgRNA responsible for identifying target DNA sequences and guiding the Cas9 enzyme to cleave double-stranded DNA [8]. Cells repair double-strand breaks through nonhomologous end joining (NHEJ) or homologous recombination (HR). The development of these techniques has greatly propelled biomedical research and therapeutic strategies [9].

Nevertheless, CRISPR-Cas9 still faces challenges in gene knock-in, especially in large animals [10]. The efficiency of gene knock-in is influenced by various factors, including sgRNA design, Cas9 expression, DNA template type and size, homologous arm length and position, cell cycle stage, and repair mechanism preferences [11]. To enhance efficiency and feasibility, this study employed an EGFP knock-in system efficiency assessment model and implemented multiple strategies, such as optimizing the CRISPR-Cas9 expression system, adjusting the form of the donor template, and using cell cycle inhibitors. Among these strategies, we explored the relatively understudied CDC25B-IN-1, a specific inhibitor targeting the CDC25B gene, which encodes a phosphatase regulating the G2/M transition of the cell cycle. In studies utilizing homologous recombination for gene knock-in, prolonging the G2/M phase can enhance knock-in efficiency, and CDC25B-IN-1 can delay cell entry into the M phase by inhibiting the activity of CDC25B [12].

This study also explored the application of gene editing technology in pig xenotransplantation, with a particular focus on the Rosa26 locus, which is important for genetically modified pig models [6,13]. Additionally, this study investigated the possibility of integrating exogenous PCMV shRNA sequences into the pig miR-17-92 gene cluster using CRISPR/Cas9 technology [14]. The miR-17-92 gene cluster encodes multiple functional miRNAs that are capable of accommodating short gene insertions without affecting their normal function [14]. By modifying the framework of the inserted siRNA, exogenous siRNA can exhibit expression patterns similar to those of endogenous miRNA [15], thus reducing the occupancy of classical safe sites and ensuring minimal disruption in future gene knock-ins in pigs. By engineering the pig genome to confer resistance against PCMV, RNAi serves as an effective gene silencing mechanism, offering a research method for enhancing pig resistance to specific viruses in xenotransplantation [16]. Although the pathogenic mechanisms of PCMV remain incompletely described in the literature, siRNA design can be based on its conserved sequences and other closely related viruses, such as human herpesvirus (HHV) [17]. Through precise gene editing and RNAi technology, this study provides a novel preventive approach against PCMV and also offers practical value in the meat market and pig farming.

## 2. Materials and Methods

All animal care and experiments complied with the guidelines of the Animal Experiment Center of Sichuan University, and this study was approved by the Ethics Committee of Sichuan University for Animal Research.

### 2.1. Establishment of the Virus Infection Model

Based on reports from other studies, 84.4% of pigs in Sichuan Province, China, are infected with PCMV. Therefore, to ensure that we could collect PCMV strains, we selected 10 pigs to test their PCMV infection status [2]. Nasal swabs were collected from 10 pigs housed at the Sichuan University WestChina Science Park. PCMV primers (Table S1) were synthesized. We utilized a commercially available virus DNA/RNA extraction kit (Tiangen Biotech, Beijing, China, DP315) to extract viral DNA from nasal swabs, where nasal mucosal tissue and buffer were mixed [18]. The viral DNA from nasal swabs was extracted using the column-based method, and the extracted DNA was stored at −20 °C for long-term preservation. The extracted DNA was then subjected to PCR amplification using TaKaRa Taq HS Perfect Mix (Takara, Chengdu, China) [19]. Following PCR amplification, gel electrophoresis was performed using Agarose (IOWEST, Madrid, Spain) prepared at a concentration of 1.5%, with Gold Viewer (Zhuangmeng, Beijing, China) added as the dye. For sample loading, 6× Loading buffer (Tsingke Biotech, Chengdu, China) was used to mix the product of PCR [20]. We selected two infected pigs and euthanized them using an overdose of Zoletil 50 (Virbac, Carros, France). Following euthanasia, we opened the thoracic cavity and retrieved the lungs. Pulmonary alveolar macrophages (PAMs) were obtained via pulmonary alveolar lavage [21]. PAMs were cocultured with porcine turbinate mucosa fibroblasts (PT-K75) (purchased from iCell Corporation), the culture medium consisted of high-glucose medium (Gibco) supplemented with 5% fetal bovine serum (Royacel, Lanzhou, China) [22], and supernatants were collected from each generation using a high-speed centrifuge (Beckman, Pasadena, CA, USA). The virus was separated and enriched using sucrose density gradient centrifugation at 20,000 rpm/min. The virus was negatively stained and observed under a transmission electron microscope (Hitachi, Tokyo, Japan).

Using the PCMV-*gB* primers with the aforementioned DNA as a template (Table S1), standard samples were synthesized and diluted in the pMD18-T vector (Takara) [23]. Fluorescence real-time quantitative PCR (Bio-Rad, Berkeley, CA, USA) was performed using RT-PCMV-*gB* primers to detect and calculate the standard curve. Porcine kidney fibroblasts (PKFs) were isolated from neonatal piglets (3 days old), and PKF was cocultured with PAMs [24]. The supernatants were collected from consecutive passages, and the virus content in the supernatant was detected using the primers used to construct the standard samples.

### 2.2. Validation and Selection of siRNAs

siRNA sequences were designed by us (Table S2) and synthesized by TSINGKE Biotech. PKFs from 5th generation continuously infected cells were plated, and transfection was performed using Lipofectamine 3000 (Invitrogen, Waltham, MA, USA) [25]. Different siRNAs at various sites were added, and after 2–4 days of culture the virus content in the supernatant was assessed.

shRNA was designed based on siRNAs at the U77 and U51 sites and synthesized by TSINGKE Biotech. Transfection efficiency was examined using an inverted fluorescence microscope (Zeiss, Lewisville, TX, USA), and transfection and virus content analysis in the supernatant were conducted using the aforementioned method [26].

siRNA targeting the U77 site was designed to interfere with the lentivirus and was synthesized by TSINGKE Biotech. Transfection efficiency was assessed using an inverted fluorescence microscope (Zeiss), and transfection and virus content analysis in the supernatant were performed using the aforementioned method.

### 2.3. Selection of the Gene Editing Site

Using the sgRNA design website (http://crispor.tefor.net/, accessed on 16 February 2023), four sgRNA sequences were designed at the Rosa26 locus (Figure S1A). The sgRNA was inserted into the PX458 plasmid, which was subsequently transfected into PKF cells using Lipofectamine 3000 (Thermo Fisher Scientific, Waltham, MA, USA) [27]. GFP-positive cells (successfully transfected cells) were collected using a flow cytometer (BD FACSAriaIII, Franklin Lakes, NJ, USA), and Sanger sequencing was performed by TSINGKE Biotech. The sequencing results were analyzed using TIDE to assess gene editing efficiency.

For the miR-17-92 locus, sgRNA sequences were designed based on the design from another report (Figure S1B) [28], and gene editing efficiency analysis was conducted following the aforementioned protocol.

### 2.4. Optimization of Gene-Targeted Integration Efficiency

This study optimized the conditions for the targeted integration of genes in pig cells. Donor plasmids were designed and synthesized based on the PUC57 plasmid backbone, which is devoid of eukaryotic promoters, contains the EGFP gene, and possesses a 400 bp left homologous arm and an 800 bp right homologous arm at the Rosa26 locus. Circular homologous recombination donors, linearized homologous recombination donors, and nonhomologous recombination donors carrying only EGFP were separately co-transfected with the PX330 plasmid constructed with sgRNA-2. The percentage of GFP-positive cells was analyzed by flow cytometry 2–4 days post-transfection, and the targeted integration efficiency was calculated [29].

Synthetic in vitro transcribed sgRNA (IVT sgRNA) was used, and the CRISPR-Cas9 system was co-transfected with a circular homologous recombination donor in plasmid, mRNA, and ribonucleoprotein (RNP) forms, followed by an analysis of targeted integration efficiency using the aforementioned method [30].

Cells were pretreated with different concentrations of CDC25B-IN-1 for 24 h. Transfection was then performed with the PX330 plasmid constructed with sgRNA-2 and a circular homologous recombination donor, followed by the analysis of targeted integration efficiency using the aforementioned method.

### 2.5. Targeted Integration of shRNA Genes

Donors for the Rosa26 and miR-17-92 loci were designed. The Rosa26 locus donor was co-expressed with both EGFP and shRNA, with a 400 bp homologous arm on the left and an 800 bp homologous arm on the right. The shRNA on the miR-17-92 locus donor was packaged based on the miR-30 scaffold, with 60 bp homologous arms added to both ends, synthesized in single-stranded DNA (ssDNA) form. After the pretreatment of PKFs with 60 μL of the CDC25B-IN-1 drug, the Cas9 mRNA, corresponding IVT sgRNA, and the aforementioned donors were co-transfected separately [31].

GFP-positive cells were picked to achieve single-cell cloning of Rosa26 locus-integrated cells. Genomic DNA was extracted from some of the single clones, followed by PCR with the primer RS1 and agarose gel electrophoresis analysis. Sequencing analysis was performed after PCR with primers RS1 and DS1 (Table S1).

Single clones of miR-17-92 locus cells were picked by microarray [24], and after they grew, genomic DNA was extracted from them. PCR was performed with primer M1, followed by agarose gel electrophoresis analysis to confirm successful integration. Sequencing analysis was subsequently conducted after PCR with primers M1, M2, and M3 (Table S1).

### 2.6. Assessment of Antiviral Capability after shRNA Knock-In

The gene-edited single clone cells were continuously cultured and passaged. Considering the long cycle of the cocultivation protocol described in Section 2.1, the supernatant from continuously infected PT-K75 was added to Rosa26 gene-edited single clone cells and wild-type (WT) cells. After 72 h, the virus content in the supernatant was detected using the aforementioned method, and the cells were collected for PCR with the primer PCMV and agarose gel electrophoresis to determine the virus content (Table S1) [32].

The supernatant from continuously infected PT-K75 cells was added to miR-17-92 gene-edited single clone cells and WT cells. The virus content in the supernatant was detected at 24 h, 48 h, and 72 h, and the cells were collected at 24 h and 72 h for PCR with the primer PCMV and agarose gel electrophoresis to detect the virus content (Table S1).

### 2.7. Safety Evaluation

Using BLAST, all potential off-target sites (OTS) for each sgRNA target sequence in the pig genome were predicted [33]. Ten potential OTS were selected for each site (Tables S3 and S4), and the primer sequences are listed in Supplementary Tables S5 and S6. Genomic DNA was extracted from the gene-edited PKFs, and PCR was performed for these twenty sites. The PCR products were collected and digested with T7E1 enzyme (NEB) for at least 30 min, followed by agarose gel electrophoresis and the sequencing of OTS [34].

Cell proliferation was assessed using the CCK-8 assay (ZhuangMeng, ZP328) on cells with gene knock-in and wild-type cells. WT and gene-edited cells were added to a 96-well plate and cultured for a certain period of time in a CO_2_ incubator. The optical density of each well was measured at 450 nm using a microplate reader at 12, 24, 36, 48, and 72 h.

### 2.8. Statistical Analysis

Statistical analysis was performed using GraphPad Prism v8.0 (GraphPad Software Inc., San Diego, CA, USA) (*t* test). All the data are presented as the mean ± SEM. Values were considered statistically significant at *p* < 0.05. The results represent three or more independent experiments.

## 3. Results

### 3.1. Establishment of the Virus Infection Model

**Figure 1:**
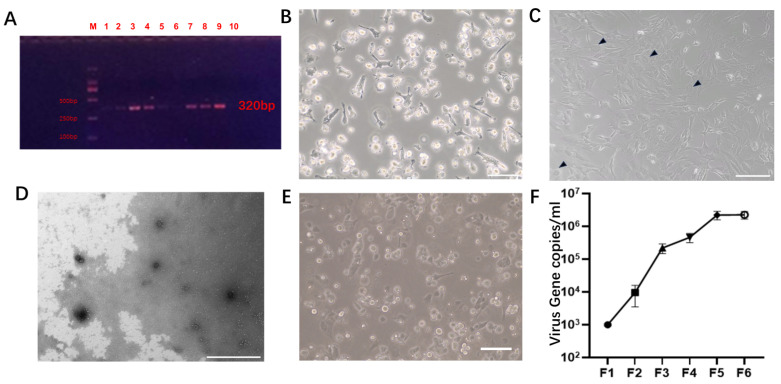
(**A**). Electrophoresis bands obtained after PCR and agarose gel electrophoresis, with samples labeled 1–10 corresponding to the tested pig IDs. M: Marker showing 100 bp, 250 bp, 500 bp, and 750 bp bands from bottom to top. (**B**). PAMs were captured at 100× magnification. Scale bar: 200 μm. (**C**). Microscopy image taken at 100× magnification showing PT-K75 and PAM coculture after four generations, with arrows pointing to giant cell lesions. Scale bar: 200 μm. (**D**). Transmission electron microscopy image of PCMV after negative staining, with arrows indicating virus particles. Scale bar: 1 μm. (**E**). Images of isolated PKFs were captured at 100× magnification. Scale bar: 200 μm. (**F**). Virus copy numbers in the supernatants of PKF cell cultures at different passages (F1–F6) after infection.

As described in other reports, nasal swabs were collected from the sampled pigs for this study and subjected to PCR amplification to detect PCMV infection [35]. All pigs were infected with PCMV (Figure 1A). We aseptically isolated PAMs from two infected pig lungs. Microscopic observation revealed an isolated PAM (Figure 1B). The coculture of PAMs with PT-K75 for four generations resulted in characteristic features of giant cell infection, such as syncytia and inclusion bodies, in PT-K75 cells (Figure 1C). Supernatants from the fourth generation coculture were selected, enriched by high-speed centrifugation, negatively stained, and observed via transmission electron microscopy. Abundant virus particles with a diameter of approximately 100–150 nm, displaying viral envelopes consistent with those described in other reports, were observed (Figure 1D). PT-K75 cells persistently infected with PCMV can be used as a vector for subsequent virus isolation.

To expand the results of this study to adult pigs in the future, considering that porcine kidney cells are both excellent nuclear donors and susceptible to PCMV infection [36], PKFs were chosen as the main cells for subsequent viral challenge experiments. PKFs were isolated from the kidneys of 3-day-old neonatal Bama miniature pigs (Figure 1E). To select the appropriate cell passage for subsequent viral challenge experiments, PKFs and PAMs were cocultured, and supernatants from the first to sixth generations of PKFs persistently infected with the virus were collected for real-time PCR analysis. The detection results were converted using a standard curve. The results showed a significant increase in virus copy number with increasing generation, reaching 2.1 × 10^6^ copies/mL and 2.2 × 10^6^ copies/mL in the fifth and sixth generations, respectively (Figure 1F). Considering that PKFs, as primary cells, may lose activity with excessive passages, the fifth generation of persistently infected PKFs was selected for siRNA screening.

### 3.2. Selection and Validation of Antiviral siRNAs

**Figure 2:**
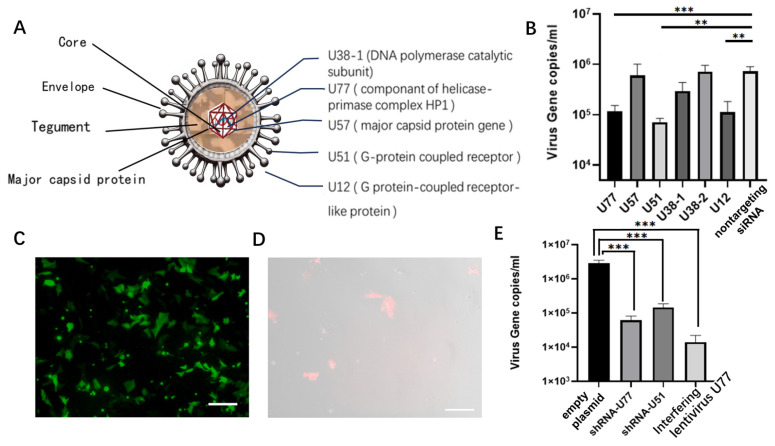
(**A**). Basic structure of the PCMV and siRNA target genes. (**B**). Quantification of virus gene copies in the supernatant of PKFs after siRNA transfection. *Y*-axis: Virus gene copies. *X*-axis: Different siRNA sequences. (**C**). PKFs were observed under an inverted fluorescence microscope after shRNA transfection at 100× magnification. Scale bar: 200 μm. (**D**). After lentivirus interference, PKFs were observed under an inverted fluorescence microscope at 200× magnification. Red and bright-field images were merged. Scale bar: 100 μm. (**E**). Quantification of virus gene copies in the supernatant of PKFs after shRNA and lentivirus transfection. shRNA1-U77, shRNA2-U51: PKFs transfected with shRNAs targeting U77 and U51, respectively. All values are the mean ± S.E.M., n = 3. ns, not significant. ** *p* < 0.01, *** *p* < 0.001.

Based on siRNA designs from reports targeting viruses similar to PCMV (such as HHV6 and HHV7), we analyzed essential and conserved sites susceptible to interference by PCMV and designed siRNAs accordingly (Figure 2A). Following the cell infection experiments, we transfected siRNAs into infected PKF cells and, after 72 h, quantified the virus levels in the cell supernatant using qPCR. Among them, siRNAs targeting U77 and U51 showed promising interference capabilities (Figure 2B).

In this study, shRNAs were synthesized based on sequences targeting U77 and U51, and they were loaded onto plasmids carrying EGFP and transfected into infected PKF cells, which demonstrated high transfection efficiency (Figure 2C). Virus levels in the supernatant were then measured (Figure 2E).

To evaluate the effectiveness of endogenous shRNA expression in interfering with the virus, the U77 sequence, which exhibited high interference efficiency, was packaged into an interfering lentivirus. This lentivirus was used to infect PKF cells for monoclonal selection (Figure 2D). Monoclonal cells were used for virus challenge experiments, and supernatants were collected. The results showed that both the exogenous expression of the U77 and U51 sequences on plasmids and the endogenous expression of the U77 sequence through the random insertion of the interference lentivirus into the genome effectively interfered with PCMV (Figure 2E), reducing virus levels to 1/20–1/170 compared to those in untreated PKFs.

### 3.3. Interference with RNA Site-Specific Integration

**Figure 3:**
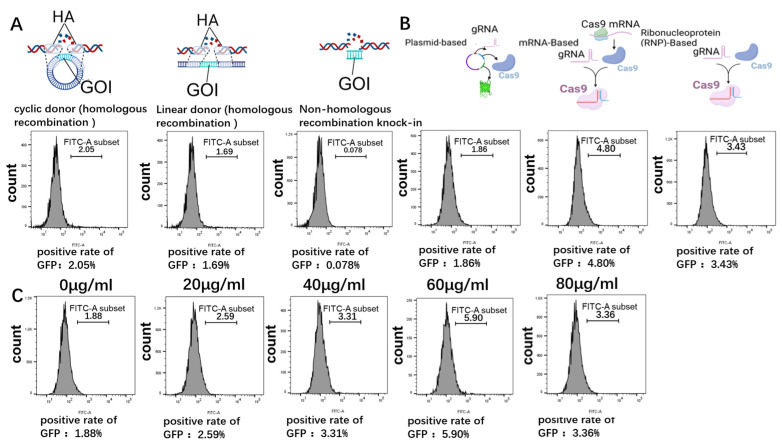
(**A**). Impact of donor form changes on gene site-specific integration efficiency. From left to right: integration with a circular donor via HR, integration with a linear donor via HR, and integration with an NHEJ donor, followed by flow cytometry analysis to detect GFP-positive cells. (**B**). Impact of CRISPR/Cas9 form changes on gene site-specific integration efficiency. From left to right: integration using plasmid form, integration using mRNA form, and integration using RNP form, followed by flow cytometry analysis to detect GFP-positive cells. (**C**). Impact of the CDC25B-IN-1 drug concentration on the gene site-specific integration efficiency. From left to right: pretreatment with 0 μg/mL, 20 μg/mL, 40 μg/mL, 60 μg/mL, or 80 μg/mL CDC25B-IN-1 for integration, followed by flow cytometry analysis to detect GFP-positive cells.

To integrate interfering RNA into the genome, appropriate integration sites and suitable donors need to be selected. The classic and validated pRosa26 site was chosen as the integration site [29,37]. This study utilized an online design tool to design four ROSA26-sgRNA sequences targeting the ROSA26 gene sequence. Subsequently, these sequences were cloned and inserted into the PX458 plasmid. However, sgRNA 3 failed to cleave due to a mutation in the original genomic locus. Consequently, sgRNA 2, which exhibited higher cleavage efficiency, was selected for further experiments (Figure S2A). An EGFP donor without a promoter was designed for exploring integration conditions, including altering donor forms, changing CRISPR/Cas9 forms, adjusting concentrations of cell cycle inhibitor drugs, and assessing their impact on gene site-specific integration efficiency. The most suitable integration conditions were selected through flow cytometric analysis. This study revealed that the efficiency of circular donor integration through homologous recombination was significantly greater than that of NHEJ integration and slightly greater than that of linear donor integration (Figure 3A). The integration efficiency of CRISPR-Cas9 using IVT sgRNA and mRNA encoding the Cas9 protein was 2.6 times greater than that of the CRISPR/Cas9 system (Figure 3B). Compared with untreated cells, cells pretreated with 60 μg/mL CDC25B-IN-1 showed a 3.1-fold increase in integration efficiency (Figure 3C).

**Figure 4:**
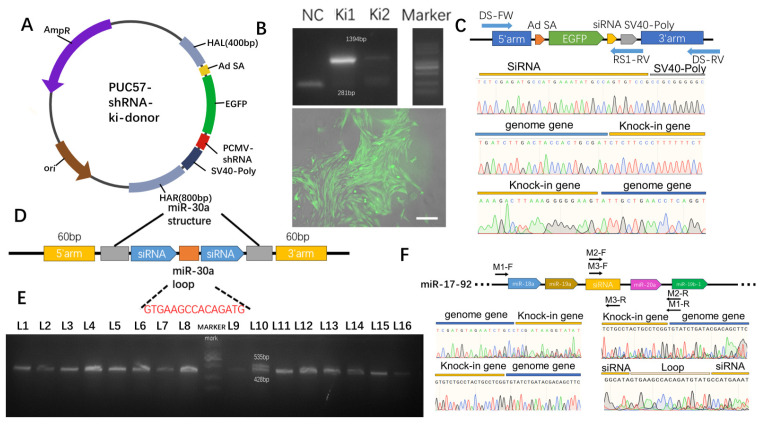
(**A**). Illustration of the Rosa26 locus integration donor. (**B**). (**Top**): Agarose gel electrophoresis to verify successful integration. NC: wild-type cells; Ki1 and Ki2: two clones with an integrated EGFP donor. The marker bands from bottom to top represent 100, 250, 500, 750, 1000, 2000, 3000, and 5000 bp, with the brightest band at 750 bp. (**Bottom**): Observation of Ki cells under an inverted fluorescence microscope at 200× magnification, with merged green and bright-field channels. Scale bar: 100 μm. (**C**). Sequencing results after integration at the Rosa26 locus. (**D**). Illustration of the miR-17-92 locus integration donor. (**E**). Agarose gel electrophoresis of PCR products to confirm successful integration. L1-L16: Clonal cells. The marker bands from bottom to top represent 100, 250, 500, 750, 1000, 2000, 3000, and 5000 bp. (**F**). Sequencing of the corresponding gene sequences after editing at the miR-17-92 locus. M1-F, M1-R; M2-F, M2-R; M3-G, and M3-R represent the corresponding sequencing primers. The sequencing results at the top left and top right correspond to M1-F and M1-R, the bottom left ones correspond to M2-F and M2-R, and the bottom right ones correspond to M3-F and M3-R.

Donor-integrating shRNA was designed (Figure 4A) and introduced using the optimized protocol mentioned above. Since the designed donor allows for the co-expression of EGFP and shRNA, the subsequent isolation of fluorescent cells was conducted. The integration of the targeted locus in single clones was assessed through agarose gel electrophoresis and inverted fluorescence microscopy. The results indicated homozygous integration in Ki1 and heterozygous integration in Ki2. Ki1 cells exhibited evident fluorescence upon passage (Figure 4B), and sequencing confirmed successful gene integration (Figure 4C).

In addition to achieving shRNA integration at the Rosa26 locus, this study selected the miR-17-92 cluster as another integration site for gene editing. The shRNA was integrated into the miR-17-92 cluster using a donor based on the miR-30 scaffold, allowing its co-expression with other miRNAs to mitigate the impact of exogenous shRNA on pig growth and development [14]. This approach also reduces occupancy at safe loci in pigs, facilitating the addition of other genetic modifications after generating gene-edited pigs. The study compared cleavage sites on the miR-17-92 cluster and selected the site with the highest efficiency, guided by sgRNA 1, for subsequent experiments (Figure S2B). The donor was synthesized based on the miR-30 scaffold (Figure 4D), and gene targeting was performed using the aforementioned method. Single clones were picked, and their gene integration status was assessed via agarose gel electrophoresis. Among them, clone L10 showed evidence of site-specific integration (Figure 4E). Subsequent sequencing confirmed successful gene integration in clone L10 (Figure 4F).

### 3.4. Validation of Antiviral Activity and Safety Assessment after Interfering with Gene Site-Specific Integration

**Figure 5:**
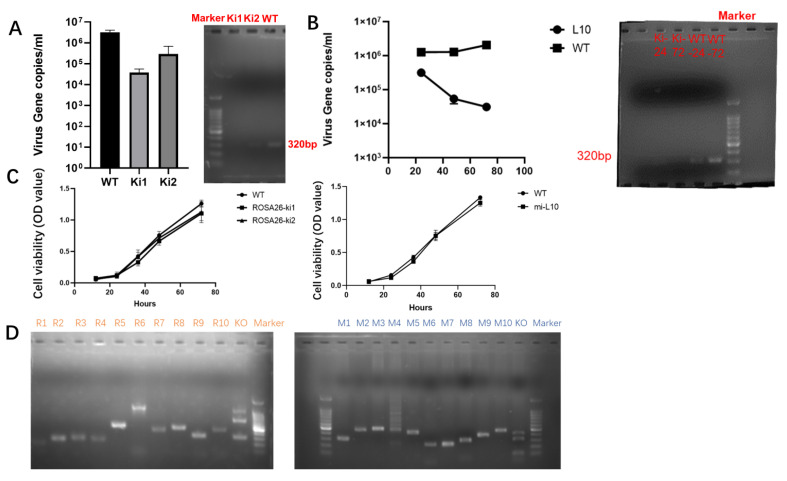
(**A**). (**Left**): Detection of viral copy numbers in the cell supernatant after interference RNA integration at the Rosa26 locus. WT: Wild-type. Ki1, Ki2: Integrated clones. (**Right**): Detection of intracellular viral DNA after interference RNA integration at the Rosa26 locus. From left to right: bands for Ki1, Ki2, and WT. The marker length ranges from 100 to 5000 bp, with the brightest band at 750 bp. (**B**). (**Left**): Changes in viral copy numbers in the cell supernatant over time after interference RNA integration at the miR-17-92 locus. WT: Wild-type. L10: Integrated clone. (**Right**): Detection of intracellular viral DNA after interference RNA integration at the miR-17-92 locus. The marker ranges from 100 to 5000 bp, with the brightest band at 750 bp. The bands from left to right represent L10 at 24 h, L10 at 72 h, WT at 24 h, and WT at 72 h. (**C**). (**Left**): CCK-8 analysis evaluating the growth and survival of Rosa26 locus-integrated cell lines. WT: wild type; ROSA26-Ki1: homozygous cells; ROSA26-Ki2: heterozygous cells. (**Right**): CCK-8 analysis evaluating the growth and survival of miR-17-92 locus-integrated cell lines. WT: Wild-type; mi-L10: Integrated gene cells. (**D**). Verification of potential off-target sites using T7E1 enzyme digestion after gene editing. In the left image, R1–R10 represent ten potential off-target sites for the Rosa26 locus. KO indicates the sgRNA site. In the right image, M1–M10 represents ten potential off-target sites for the miR-17-92 locus. KO indicates the sgRNA site. The marker ranges from 100 to 5000 bp, with the brightest band at 750 bp.

This study conducted sequential assessments of the antiviral replication capabilities of cells following integration at the Rosa26 and miR-17-92 loci. In the antiviral activity assay at the Rosa26 locus, the experimental results revealed that both Ki1 and Ki2 exhibited robust virus inhibition capabilities, with Ki1 showing homozygous integration displaying a viral content in the supernatant of only 3.8 × 10^4^ cells, reducing the viral content to approximately 1/68 of that in wild-type cells, which was greater than that observed in the heterozygous integration cells (Ki2). Additionally, DNA extraction and agarose gel electrophoresis of Ki1, Ki2, and WT cells revealed a significant reduction in the intracellular viral content after gene editing, highlighting the promising potential of Rosa26 locus integration for anti-PCMV shRNA integration (Figure 5A).

To assess antiviral activity at the miR-17-92 locus, a clonal cell line (L10) with integration was selected and a virus challenge experiment was conducted simultaneously with wild-type cells at different time points to measure the viral content in the cell supernatant. The results showed that the clonal cells integrated at the miR-17-92 locus exhibited high viral interference capabilities, with a viral content of only 3.1 × 10^4^ in the supernatant at 72 h, reducing the viral content to approximately 1/66 that of wild-type cells. Furthermore, DNA extraction and agarose gel electrophoresis of L10 and WT cells at 24 and 72 h revealed a significant reduction in the intracellular viral content at 24 h post-gene editing, with a much lower viral content than that of WT cells at 72 h, indicating the potential of the miR-17-92 locus for expressing RNAi to interfere with PCMV (Figure 5B).

The morphology of the PT-K75 cell line significantly changes after PCMV infection. The sequencing of the miR-17-92 integration site in the PT-K75 cell line revealed its relative conservation without mutations (Figure S3A). The integration at the miR-17-92 locus in PT-K75 cells was confirmed through clonal selection and sequencing analysis (Figure S3B). The results of the viral challenge experiment revealed that after repeated passages, the PT-K75 cell line with knocked-in shRNA maintained a stable morphology relative to that of the wild-type cell line (Figure S3C). Even after repeated passages, the knock-in of shRNA remained effective, indicating its sustained functionality.

Proliferation assays were conducted to evaluate the growth capacity of cells after integration at the Rosa26 and miR-17-92 loci to avoid damaging cell proliferation. After gene editing, the cells were cultured simultaneously with wild-type cells, and cell proliferation was measured using the CCK-8 assay. The results showed no significant differences in cell proliferation between cells integrated at the Rosa26 or miR-17-92 locus and wild-type cells (Figure 5C).

Subsequent off-target analysis was conducted on these two gene editing loci. Ten potential off-target sites and corresponding primers were retrieved and designed for the Rosa26 and miR-17-92 loci based on NCBI data. PCR analysis of these off-target sites followed by T7E1 enzyme digestion revealed no mutations at potential off-target sites (Figure 5D). Sanger sequencing of the potential off-target sites at the Rosa26 locus confirmed the absence of off-target mutations (Figure S4 and Figure 5).

## 4. Discussion

In recent years, PCMV has become an important research focus due to its adverse effects on xenotransplantation [4]. However, compared to other viruses, there has been relatively less in vitro research on the mechanisms of action, prevention, and control of PCMV. Therefore, investigating methods for preventing and treating PCMV is highly important for advancing the development of xenotransplantation. In this study, PCMV infection was detected in pigs through PCR, and PAMs were extracted from infected pigs’ lungs. The PT-K75 cell line was utilized to establish a model for sustained virus infection and production, and the presence of the virus was confirmed morphologically and through transmission electron microscopy, along with the construction of a standard curve for PCMV. PKFs were used as a model for gene editing and subsequent infection experiments, and the replication status of PKFs at different passages after infection was evaluated, with the fifth passage of PKFs selected as the target for siRNA screening. However, it is worth noting that PKF is advantageous due to its susceptibility to PCMV infection and its suitability for somatic cell nuclear transfer (SCNT). When used only for SCNT, porcine fetal fibroblasts (PFFs) can also serve as suitable cells for somatic cell nuclear transfer [38].

This study screened and validated siRNA targets and sequences that effectively inhibit PCMV replication. Based on the genomic sequences of PCMV and siRNA reported in the literature for similar viruses, five sites were selected—U77, U57, U51, U38, and U12—and transfected into cells. Additionally, the siRNA sequences of U77 and U51 were inserted into shRNA plasmids, which were subsequently transfected into PKF cell lines via the same method. Furthermore, interference lentivirus technology was utilized to integrate U77-shRNA into the chromosomes of PKF cells to detect the effective integration of exogenous shRNA into their genomes, and the results were compared with those of cells transfected with shRNA and wild-type cells. The main results indicate that siRNAs targeting three sites—U77 (a component of the helicase-primase complex HP1), U51 (a G protein-coupled receptor), and U12 (a G protein-coupled receptor-like protein)—significantly reduce the copy number of PCMV. These findings suggested that these three sites are important and conserved targets for PCMV replication, with the G protein-coupled receptor being of interest in murine cytomegalovirus and the component of the helicase-primase complex being associated with the lytic DNA replication machinery of herpesviruses [39,40]. Interfering with their expression or function can effectively inhibit virus replication, providing new insights and methods for the prevention and treatment of PCMV infection. The interference lentivirus carrying U77-shRNA continuously inhibited PCMV replication even more effectively than the shRNA alone. This indicates that stable and persistent antiviral effects can be achieved by interfering with the endogenous expression of exogenous shRNA via lentivirus interference, providing experimental evidence for the rationality of the CRISPR-Cas9-mediated targeted integration of shRNA. In the future, leveraging PCMV infection models and PCMV content detection models for larger-scale screening could consider other potential virus gene target sites, such as U14, U54, and U69, to explore the virus—cell interaction mechanisms of PCMV.

CRISPR/Cas9 technology has demonstrated high efficiency and convenience in gene knockout, but its application in gene knock-in has been limited by its lower efficiency in homologous recombination repair. In this study, we utilized the EGFP gene as a reporter gene to explore and optimize the conditions and parameters of CRISPR/Cas9 gene knock-in technology through the detection of its fluorescent signal. We demonstrated that the ROSA26 locus is an ideal target site for both gene knock-in and knockout. Our sgRNA design method can also serve as a reference for other gene editing projects. Several key factors affecting gene knock-in efficiency were identified, including the form of the donor, the delivery method of CRISPR/Cas9, and the use of cell cycle inhibitors. Our optimization methods significantly improved the success rate of gene knock-in, with the use of mRNA as the delivery method for CRISPR/Cas9 and the use of 60 μg/mL CDC25B-IN-1 resulting in gene knock-in positive rates of 4.80% and 5.90%, respectively, thus providing more possibilities for the application of gene editing. Additionally, the EGFP knock-in model shows potential for further research, including the optimization of experimental protocols based on homologous arm length and the use of other cell cycle inhibitors [29].

In xenotransplantation from pigs to humans, the elimination of PCMV from pig organs is especially crucial for biosafety [41,42]. The approach proposed in this study differs from recent solutions for PCMV, which have focused on pre-transplantation testing and post-transplantation drug control. Instead, we used endogenously expressed viral siRNA to address the problem of viral infection at its source. This provided a potential solution for clearing viruses from pig cells and facilitating pig organ transplantation, contributing to the health and welfare of pigs, as well as the application of pigs as biomedical models. This study simultaneously targeted the ROSA26 locus and miR-17-92 locus, aiming to achieve effective interference with the PCMV while not affecting the normal expression of genes. Ultimately, we obtained PKFs with endogenously expressed siRNAs, which can be directly used as donors for nuclear transplantation.

## 5. Conclusions

This study demonstrated the strong interference capability of siRNAs designed for the U77, U51, and U12 gene loci against PCMV. It was verified that pretreating cells with 60 μg/mL CDC25B-IN-1, as well as using mRNA or RNP forms of the CRISPR-Cas9 system, can effectively enhance gene knock-in efficiency. Furthermore, this study conducted a knock-in of shRNA at the Rosa26 and miR-17-92 loci, confirming successful integration. Knocking in shRNAs at these two loci can exert effective antiviral effects without affecting cell proliferation or causing off-target effects.

## Data Availability

The data presented in this study can be found in the manuscript.

## Supplementary Materials

The following supporting information can be downloaded at https://www.mdpi.com/article/10.3390/microorganisms12040837/s1, Figure S1. (A). pROSA26 locus targeting and sgRNA selection, where sequences in different colors represent different sgRNA target sites, with arrow direction indicating the direction of the sgRNA sequence; (B). sgRNA selection on the miR-17-92 cluster, where the sgRNA1 sequence is in the forward direction and the sgRNA2 sequence is in the reverse direction. Figure S2. After knock-in, the sgRNA editing efficiency was analyzed through Sanger sequencing and TIDE analysis. The upper part displays the peak charts of Sanger sequencing results, while the lower part shows the TIDE analysis. (A). From left to right: sgRNA1, sgRNA2, sgRNA4 targeting the Rosa26 locus. (B). From left to right: sgRNA1, sgRNA2 targeting the miR-17-92 cluster. Figure S3. (A). The upper panel displays the sgRNA sequences targeting the miR-17-92 cluster in PT-K75 cells, while the lower panel shows the sgRNA sequences targeting the miR-17-92 cluster in PKF cells. (B). Gene sequencing profiles of the miR-17-92 cluster site in PT-K75 cells post-targeted integration. (C). Morphological changes of PT-K75 cells post-targeted integration at the miR-17-92 cluster site and wild-type PT-K75 cells upon viral challenge at different passages. The left column represents edited cells, while the right column depicts wild-type cells. From top to bottom: before viral challenge, after 3 passages, 4 passages, and 5 passages post-viral challenge. Scale bar: 200 μm. Figure S4. Sequencing analysis of PCR products at potential off-target sites of the Rosa26 locus. Red arrows indicate putative cleavage sites. Figure S5. Sequencing analysis of PCR products at potential off-target sites of the miR-17-92 locus. Red arrows indicate putative cleavage sites. Table S1. Partial primer sequences used for agarose gel electrophoresis and sequencing. Table S2. SiRNA targeting sequence. Table S3. OTS design for Rosa26 locus. Table S4. OTS design for miR-17-92 locus. Table S5. Primer design for OTS detection at the Rosa26 locus. Table S6. Primer design for OTS detection at the miR-17-92 locus.
