# Supplementary material for: Targeted Integration of siRNA against Porcine Cytomegalovirus (PCMV) Enhances the Resistance of Porcine Cells to PCMV
Source: Microorganisms. 2024 Apr 22;12(4):837. doi: 10.3390/microorganisms12040837 (PMC11051760; doi:10.3390/microorganisms12040837)
Supplement: Supplementary file 1 [file microorganisms-12-00837-s001.zip › support - figure.pdf]

Supplementary information

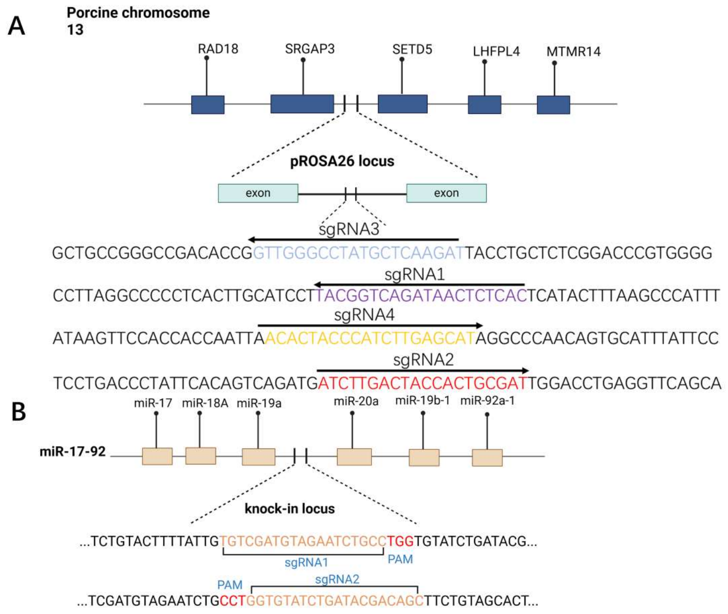

Figure S1. (A). pROSA26 locus targeting and sgRNA selection, where sequences in different colors represent different sgRNA target sites, with arrow direction indicating the direction of the sgRNA sequence; (B). sgRNA selection on the miR-17-92 cluster, where the sgRNA1 sequence is in the forward direction and the sgRNA2 sequence is in the reverse direction.

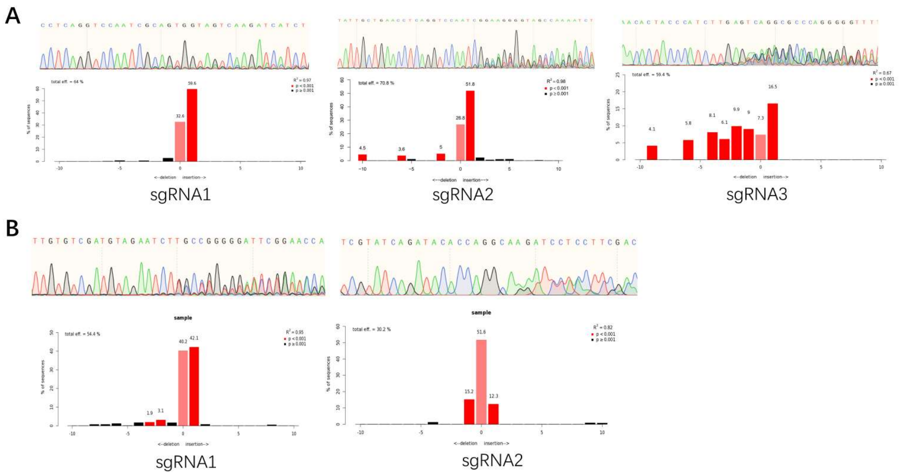

Figure S2. After knock-in, the sgRNA editing efficiency was analyzed through Sanger sequencing and TIDE analysis. The upper part displays the peak charts of Sanger sequencing results, while the lower part shows the TIDE analysis. (A). From left to right: sgRNA1, sgRNA2, sgRNA4 targeting the Rosa26 locus. (B). From left to right: sgRNA1, sgRNA2 targeting the miR-17-92 cluster.

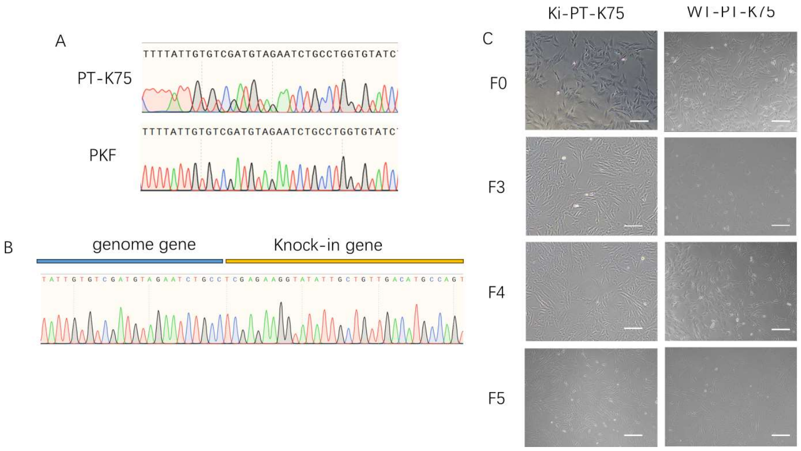

Figure S3. (A). The upper panel displays the sgRNA sequences targeting the miR-17-92 cluster in PT-K75 cells, while the lower panel shows the sgRNA sequences targeting the miR-17-92 cluster in PKF cells. (B). Gene sequencing profiles of the miR-17-92 cluster site in PT-K75 cells post-targeted integration. (C). Morphological changes of PT-K75 cells post-targeted integration at the miR-17-92 cluster site and wild-type PT-K75 cells upon viral challenge at different passages. The left column represents edited cells, while the right column depicts wild-type cells. From top to bottom: before viral challenge, after 3 passages, 4 passages, and 5 passages post-viral challenge. Scale bar: 200μm.

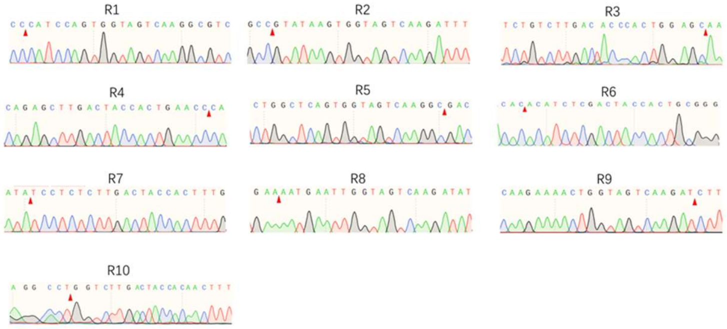

Figure S4. Sequencing analysis of PCR products at potential off-target sites of the Rosa26 locus. Red arrows indicate putative cleavage sites.

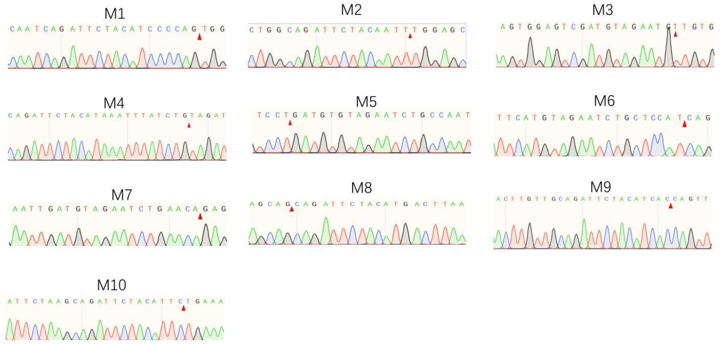

Figure S5. Sequencing analysis of PCR products at potential off-target sites of the miR-17-92 locus. Red arrows indicate putative cleavage sites.
