# Supplementary material for: Targeted Integration of siRNA against Porcine Cytomegalovirus (PCMV) Enhances the Resistance of Porcine Cells to PCMV
Source: Microorganisms. 2024 Apr 22;12(4):837. doi: 10.3390/microorganisms12040837 (PMC11051760; doi:10.3390/microorganisms12040837)
Supplement: Supplementary file 1 [file microorganisms-12-00837-s001.zip › support -table.pdf]

Supplementary information

Table S1. Partial primer sequences used for agarose gel electrophoresis and sequencing

Gene Target	sequences	Product length
PCMV	F: ACGGGGATCGACGAGAAAG<br>R: GAAGAGAAAGGAAGTGAAGG	320
PCMV-gB	F: TTCCTGTAGATGAAGCTGGAT<br>R: AGGGATAGTTTTCTTTGGTCCA	443
RT-PCMV-gB	F: TTCCGCTGCCGAGATTAGAAACCA<br>R: CTCAACCGATTCACCTGTACCGAGT	268
DS1	F: CTGAGTTTTACAGTCATCCC<br>R: TACCAAACATACAAAAGAACTG	1203
RS1	F: CCACCACCAATTAACACTACC<br>R: TATTAGCATTACGGCAACTGAG	281（After integration: 1394）
M1	F: GGTCTGCTGGTGTTGAGTG<br>R: CCGAGGCTGGAGTTCTACA	428（After integration: 535）
M2	F: CATAGTGAAGCCACAGAT<br>R: CCGAGGCTGGAGTTCTACA	174
M3	F: CATAGTGAAGCCACAGAT<br>R: CACCGAGGCAGTAGGCAGACACT	60

*F: Forward primer, R: Reverse primer

Table S2. SiRNA targeting sequence

siRNA	sequences	Reverse sequences
U77	CGGACACUGGCAUAUUUCAUGGCAU	AUGCCAUGAAAUAUGCCAGUGUCCG
U57	GAGGCCAUACUCGGCAUCUACUGUA	UACAGUAGAUGCCGAGUAUGGCCUC
U51	GGAUACUGCGUUCUACAUA	UAUGUAGAACGCAGUAUCC
U38-1	CCAGAUUCUACAUCGAUAA	UUAUCGAUGUAGAAUCUGG
U38-2	CAACUACAGACAUCAUGUA	UACAUGAUGUCUGUAGUUG
U12	GAUGCAGACUUUGCGAUAA	UUAUCGCAAAGUCUGCAUC
nontargeting siRNA	UUCUCCGAACGUGUCACGU	ACGUGACACGUUCGGAGAA

Table S3. OTS design for Rosa26 locus

OTS and sgRNA (pRosa26)	sequences

sgRNA	ATCTTGACTA CCACTGCGAT
R1	GCCTTGACTA CCACTGGATG
R2	ATCTTGACTA CCACTTATAC
R3	GTCTTGACAC CCACTGGAGC
R4	AGCTTGACTA CCACTGAACC
R5	GGCTCAGTGG TAGTCAAGGC
R6	CGCAGTGGTA GTCGAGATGT
R7	AAGTGGTAGT CAAGAGAGGA
R8	ATCTTGACTA CCAATTCATT
R9	AGAAAACTGG TAGTCAAGAT
R10	GGTCTTGACT ACCACAACTT

Table S4. OTS design for miR-17-92 locus

OTS and sgRNA (miR-17-92)	sequences
sgRNA	TGTCGATGTA GAATCTGCC
M1	ATCAGATTCT ACATCCCCAG
M2	ACTGGCAGA TTCTACAATT
M3	AGTGGAGTCG ATGTAGAATG
M4	ATTCTACATA AATTTATCTG
M5	GATGTGTAGA ATCTGCCAAT
M6	CATGTAGAAT CTGCTCCAT
M7	TTGATGTAGA ATCTGAACA
M8	TTAAGTCATG TAGAATCTG
M9	TGTTGCAGAT TCTACATCAC
M10	TCTAAGCAGA TTCTACATTC

Table S5. Primer design for OTS detection at the Rosa26 locus

OTS (Rosa26)	Forward primer	Reverse primer
OTS-R1	GTATTACAGTTGAGGTCCACGATT	TGTAACCTGCTGGTACTCATTCT
OTS-R2	ACTTGTGCTCCAGTTGAATCC	TGCTGAGGCTTGCTGACA
OTS-R3	TGTGAATCTCTATGGTCGTAGG	TGCTCCAGTGGTAGTCAAGA
OTS-R4	TCTCCTACTCCGTGCTGTT	ACTATGCTGACTGTTAGAATGG
OTS-R5	AAGAAGGAGGAGGAAGAGATGG	GCACAGACATACAGAGGAATGG
OTS-R6	GTCTGTCTACGCTGGTCCGA	TGCCTTGGCTCTCAGGTCTC
OTS-R7	GTCACAAGTGCGGCTCTAA	AGTACATGGAACACAGCAGATT

OTS-R8	GCCGATACAAGTTACCAGACA	GCACAGGAAGGTGAGAAGG
OTS-R9	AGTGGCATCAGTGACCTATTCC	GCAGTTCCTCTCGCATCTCTAT
OTS-R10	TCCGTTCCGTTGATGTCCATAT	CGGCAGGAAGTCCAATAAGGT

Table S6. Primer design for OTS detection at the miR-17-92 locus

**OTS (miR-17-92)**	**Forward primer**	**Reverse primer**
OTS-M1	GACATGACATGCGAGAGGATT	GACACATCACAGGAGAGGAAC
OTS-M2	AAGGCAGAGTCCTACCTACTAA	GGGATTTCTGGCATACATTAGAG
OTS-M3	TTGTGATGCTGCTGCTACC	TCTTCTTCTGGCTGTAACTGTT
OTS-M4	CAGCAACGCCAGATCCTTA	GGTGGTCGTCAGAACTCTC
OTS-M5	CACACTCGGCATCCTAATCTA	TCCGTGTTCAGTGATGTCATT
OTS-M6	TGTGGATGTTAGCGACTTGGA	TTGGACTGATGGAGCAGATTCT
OTS-M7	CTCTCCACTCTGGTACACTGA	GCCACGAAGACAAGTTCTGT
OTS-M8	CACTCTTCCGTCCTCCTCAT	TGCCATCTGCTCCTACAACT
OTS-M9	AGAGCACAAGGCAGGGATAG	TCCAGCCAATGAGACCACTT
OTS-M10	GCAGAGGTTGGTTCATTAGGTT	TAAGCCACGGTGTAATTAGCAA
